# Recovery from an acute systemic and central LPS-inflammation challenge is affected by mouse sex and genetic background

**DOI:** 10.1371/journal.pone.0201375

**Published:** 2018-08-22

**Authors:** Gabriela Meneses, Marcos Rosetti, Alejandro Espinosa, Alejandra Florentino, Marcel Bautista, Georgina Díaz, Guillermo Olvera, Brandon Bárcena, Agnes Fleury, Laura Adalid-Peralta, Edmundo Lamoyi, Gladis Fragoso, Edda Sciutto

**Affiliations:** 1 Departamento de Inmunología, Instituto de Investigaciones Biomédicas, Universidad Nacional Autónoma de México; Ciudad de México, México; 2 Departamento de Biología Celular y Fisiología, Instituto de Investigaciones Biomédicas, Universidad Nacional Autónoma de México; Ciudad de México, México; 3 Departamento de Medicina Genómica y Toxicología Ambiental, Unidad Periférica del Instituto de Investigaciones Biomédicas, Universidad Nacional Autónoma de México en el Instituto Nacional de Neurología y Neurocirugía Dr. Manuel Velasco Suárez, Ciudad de México, México; 4 Unidad Periférica del Instituto de Investigaciones Biomédicas, Universidad Nacional Autónoma de México en el Instituto Nacional de Neurología y Neurocirugía Dr. Manuel Velasco Suárez, Ciudad de México, México; Universidade de Sao Paulo, BRAZIL

## Abstract

Genetic and sexual factors influence the prevalence and the pathogenesis of many inflammatory disorders. In this study their relevance on the peripheral and central inflammatory status induced by a peripheral injection of lipopolysaccharide (LPS) was evaluated. BALB/c and CD-1 male and female mice were intraperitoneally injected with LPS. Spleens and brains were collected 2 and 72 hours later to study the levels of IL-6, TNF-α and IL-1β. Percentage of microglia and astrocytes was determined in the cortex and hippocampus. Locomotor activity was registered before and during the 72 hours after LPS-treatment. Two hours after LPS-injection, a peripheral increase of the three cytokines was found. In brains, LPS increased TNF-α only in males with higher levels in CD-1 than BALB/c. IL-1β increased only in CD-1 males. IL-6 increased in both strains with lower levels in BALB/c females. Peripheral and central levels of cytokines decline 72 hrs after LPS-treatment whilst a significantly increase of Iba-1 expression was detected. A dramatic drop of the locomotor activity was observed immediately after LPS injection. Our results show that acute systemic administration of LPS leads to peripheral and central increase of pro-inflammatory cytokines and microglia activation, in a strain and sex dependent manner.

## Introduction

Bacterial cell wall components are capable of activating the innate immune system, as evidenced by an inflammatory response. Among them, the endotoxin lipopolysaccharide (LPS), a cell wall component of Gram-negative bacteria, is one of the most extensively studied [1]. LPS has been experimentally used to promote an acute and chronic peripheral inflammation resembling pathological states [2–5]. This inflammatory response is triggered by the recognition of LPS by TLR4 and CD14, both of which are constitutive receptors expressed in peripheral antigen presenting cells. LPS binding promotes the activation of nuclear factor κB (NF-κB) signaling pathway [6–8]. TLR4 is also present in mouse perivascular macrophages, microglia, astrocytes and in trigeminal and nodose ganglia, as well as in other resident cells of the central nervous system (CNS) [9, 10].

When peripherally administered, LPS induces an exacerbated CNS inflammatory response which involves the activation of microglia and perivascular macrophages as well as an increased expression of several pro-inflammatory cytokines, IL-1β, IL-6 and TNFα, all messengers for immune-to-brain signaling [11, 12]. A peripheral increase of pro-inflammatory cytokines can also activate brain endothelial cells, modifying the blood–brain barrier (BBB) permeability which in turn facilitates immune cells infiltration into the brain parenchyma [13]. In addition, increased peripheral cytokines can trigger the cholinergic pathway through afferent fibers of the vagus nerve [14].

The magnitude and extension of the inflammatory response induced depends on the dose, the site and number of LPS injections employed [15–17]. Under a single low dose of LPS, inflammation is a reversible phenomenon. However, using higher and/or multiple repetitive doses of LPS peripherally injected, it is possible to induce a chronic neuroinflammatory status that leads to neurodegeneration [18].

Since the prevalence and intensity of many inflammatory disorders appear to be influenced by sex and genetic background [19], the current study reports the effects of a single peripheral injection of LPS on the peripheral and central inflammatory status (TNF-α, IL-1β, IL-6, microglia and astrocytes activation) and their impact on the locomotor activity in male and female mice of a syngenic (BALB/c) and an outbred (CD-1) strains. The relation between the neuroinflammation induced and the locomotor activities is discussed.

## Material and methods

### Mice

We tested sexually mature (6–8 weeks) male and female mice from the syngenic BALB/cAnN (herein referred as BALB/c) and outbred CD-1 strains, originally purchased from Charles River Laboratories (Wilmington, MA, USA) and subsequently bred at Instituto de Investigaciones Biomédicas, Universidad Nacional Autónoma de México (UNAM). Mice were divided into groups of five, and kept in polysulfone boxes with food and water ad libitum before and during the experiments. Mouse housing was held at 22 ± 3°C with a 12:12 h light-dark cycle. This study protocol was approved by the Institutional Committee for the Use and Care of Laboratory Animals of the Instituto de Investigaciones Biomédicas, UNAM (approval number ID 232). All housing and experimental procedures were conducted under the guidelines established by this Committee. Every effort was made, especially during LPS challenge and euthanasia, to minimize animal suffering and stress.

### Experimental design

Male and female mice of each strain were randomly divided into three groups of five mice each. Two groups were i.p. injected with a single dose of either endotoxin-free saline (injectable solution CS, PiSA Laboratories, México) or LPS (1 mg/kg; *Escherichia coli* serotype 0111:B4, Sigma, Saint Louis, MO, USA). A third group of mice received no treatment. Two and 72 hours after treatment, cytokine levels (TNF-α, IL-1β and IL-6) were evaluated and the expression of ionized calcium-binding adaptor molecule 1 (Iba-1) and glial fibrillary acidic protein (GFAP), which are specifically expressed in microglia and astrocytes, respectively, was determined by immunofluorescence analysis. For locomotion studies, mice were conditioned 72 h before LPS administration, and their behavior was recorded for 2 minutes every 4 hours for a total of 72 hours after LPS.

### Preparation of tissue extracts

Two and 72 hours after LPS treatment, mice were deeply anesthetized with a lethal intraperitoneal injection of 90–100 mg ketamine and 10 mg xylazine per kg. Brains and spleens were rapidly removed, kept on ice and homogenized in 3 volumes of lysis buffer (PBS 1X, supplemented with 1% of phenylmethylsulfonyl fluoride at 1mM, Sigma Aldrich P7626) at 4°C. Lysates were then centrifuged at 8124 X *g* for 15 min at 4°C; supernatants were collected and stored at −80°C until used. Proteins in the soluble extract were measured using the Lowry method [20].

### Cytokine Enzyme-Linked Immunosorbent Assay (ELISA)

Commercial kits were employed to quantify in tissue extracts the pro-inflammatory mouse cytokines IL-1β, IL-6 and TNF-α (all from BioLegend, San Diego, CA, USA), following the supplier's instructions. Sandwich ELISAs were performed in 96-well, flat-bottom microtiter plates (Nunc-Immuno Plate Maxisorp, Rosekilde, Denmark). Microplates were coated with the capture antibody for 18 hrs at 4°C. After washing with PBS–Tween–20 (0.05%) and blocking for 30 min at room temperature with 2% PBS–BSA, plates were incubated for a further 18 hrs, at 4°C with antibody standards and samples, washed three times and incubated for 1 hr with the detection antibody at room temperature. Bound detection antibodies were identified using 1:10,000 diluted streptavidin–phosphatase conjugate (BD Pharmingen) and p-nitrophenylphosphate (Sigma) as substrate. Optical density was read at 450 nm after 30 min of incubation. Assay sensitivity was measured in pg/mL.

### Immunofluorescence (IFC) analysis

Histological studies were performed by immunofluorescence following the protocol previously described [21]. Briefly, each brain was fixed in 4% buffered paraformaldehyde at 4°C overnight. Free-floating 30 mm-thick mouse brain sections were processed as previously described [21]. After antigen retrieval by incubating in citrate buffer (0.01 M citric acid, 0.05% Tween 20, pH 6.0) at 70°C for 50 min, samples were washed thoroughly several times with Tris-buffered saline (TBS) and blocked with a solution of 2% immunoglobulin (IgG-free albumin; Sigma, Saint Louis, MO, USA) in TBS for 20 min at room temperature. Brain sections were then incubated overnight at 4°C with either rabbit anti-GFAP (Invitrogen, Carlsbad, CA, USA) or anti-Iba-1 (Wako Chemicals, Inc., Richmond, VA, USA), in TBS–2% BSA to detect astrocytes and microglia, respectively. After washing, sections were incubated for 1 h at room temperature with AlexaFluor 594 goat anti-rabbit IgG (Molecular Probes, Eugene, OR, USA) diluted in TBS–2% BSA. Primary antibody was substituted by TBS-2% BSA in negative controls that were carried out in parallel. Samples were mounted onto glass slides in Vectashield medium (Vector Laboratories, Burlingame, CA, USA) containing 4’,6-diamidino-2-phenylindole (DAPI) for nuclei imaging. Stained slides were analyzed using an Eclipse Ci-L/Ci-S Upright Microscope equipped with a DP71 digital camera, DP-Manager and DP-Controller software (Olympus, Center Valley, PA, USA). Slides were previewed and optimum gain and exposure time (1/2.3 and 1/15 seconds for Iba-1 and GFAP, respectively) were calculated for a standardized data acquisition. Regions of the hippocampus (*Cornu Ammonis* CA1 and CA2) and two regions of the cortex (R1 and R2) as previously illustrated elsewhere [21], were selected and quantifying pixel areas was performed with color threshold tool of ImageJ software (National Institute of Health, Bethesda, MD, USA; https://imagej.nih.gov/ij/index.html). The same software was employed to estimate the percentage of Iba-1 or GFAP in the mentioned regions.

### Locomotor activity

In order to quantify the effect of LPS on the locomotor activity, five mice inside the polysulfone boxes were filmed and scored. Experimenters filmed the mice for two min every four hrs, starting four hours before treatment and for the 72 hrs following i.p. LPS or ISS administration or no intervention (controls), producing a total of 25 tests of locomotion for each group of mice. Mice were filmed from above, using a video camera on a tripod and framing the entire surface of the cage. During the dark photoperiod, infrared illumination was used to film the mice. The locomotor activity of each mouse was scored by dividing the area with bedding material (Sani chip, Harlan) into 6 squares of equal size by overlapping a grid onto the video. Locomotor activity was quantified as the number of quadrants crossed in two minutes. A mouse was considered that crossed into a new quadrant when all the mouse legs were inside it. Behavioral observations were performed by blinded observers. Observers obtained an inter-rater reliability rating of 0.87, as measured using Spearman's rho for pairwise comparisons. To evaluate recuperation times, we analyzed locomotion scores from two independent experiments with 10 mice (5 male, 5 female) in each treatment group. We calculated the percent change of frequency of movement of LPS mice at the time when more activity was present (24 hrs), compared to that of ISS-treated and controls. The large impact of LPS on behavioral measures and the consistency of locomotor activity in the light and dark photo-periods led us to choose a small sample size and still achieve a significant effect. At the end of the experiment all mice were euthanized.

### Statistical analysis

The level of cytokines measured are expressed as the means ±SD, resulting from four independent experiments with three to 5 mice in each treatment group. Sample size was chosen based on a pilot study. Significant differences between groups were determined by a two-tailed unpaired t-test with Welch´s correction performed on the basis of unequal variance, while differences between groups of means ±SD of the percent of pixels/μm2 of the Iba-1 or the GFAP expression were compared a Kruskal-Wallis followed by Mann-Whitney test. Tests were carried out using GraphPad Prism® 5.0 (GraphPad Software, San Diego, CA, USA).

To evaluate recuperation times, the locomotion scores for each group of mice at times of peak activity, corresponding to the middle of the dark photoperiod (i.e. 24, 48, 72 and 92 hrs) were compared. Comparisons were done by sex (male vs female), strain (BALB/c vs CD-1) and treatment (LPS vs SSI+C). All comparisons were done using Student t-tests with Holm's correction for multiple comparisons, which were performed in R statistical software [22]. Statistical significance for all tests was considered when p values were lower than 0.05.

## Results

### Effects of systemic LPS administration on peripheral and central levels of pro-inflammatory cytokines

Based on the course of neuroinflammation reported in other studies [23–25], cytokine levels in spleens and brains of male and female BALB/c and CD-1 were measured 2 and 72 hrs after i.p. inoculation of 1 mg/kg of LPS.

**Fig 1:**
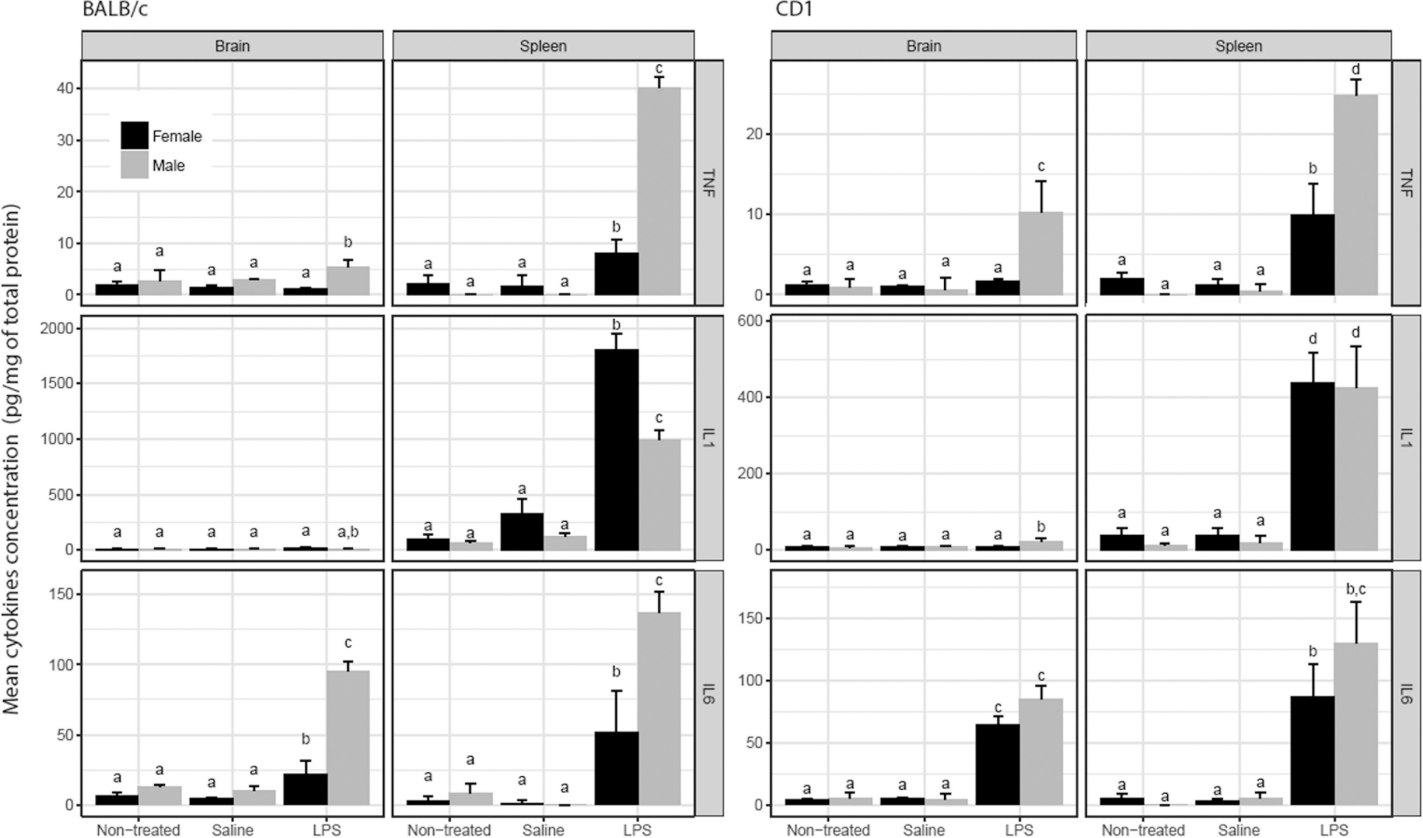
Cytokine levels in CD-1 and BALB/c male and female mice two hours after LPS administration. †Mean ±SD of the level of cytokines (pg/mg of total protein) in spleen and brains of non-treated, saline or LPS-treated in groups of three to five BALB/c and CD-1 male and female mice. Different literals indicates statistically significant (P<0.05) differences between values of each column using an unpaired t test with Welch’s corrections.

In the periphery, 2 hours after LPS injection, TNF-α, IL-1β and IL-6 were found to be significantly increased. Male mice of both strains exhibited higher TNF-α and IL-6 levels than female (P<0.05). LPS-treated BALB/c male mice had the highest levels of TNF-α (Fig 1). Female BALB/c mice expressed more IL-1β than male. In the central nervous system, LPS significantly increased levels of TNF-α but only in males and with higher levels in CD-1 than BALB/c. In brains, whilst IL-1β is only significantly increased in CD-1 males, IL-6 increases in male and female of both strains, with lower levels in BALB/c females.

Peripheral and central levels of the three cytokines showed a steady decline 72 hrs after LPS-treatment (see S1 Fig). Only IL-1 β remained significantly increased in the periphery in BALB/c male mice and CD1 female throughout the 72 hr period.

### Effects of systemic LPS administration on the percentage of GFAP1 and Iba-1 brain cells 2 and 72 hours after administration of LPS

**Fig 2:**
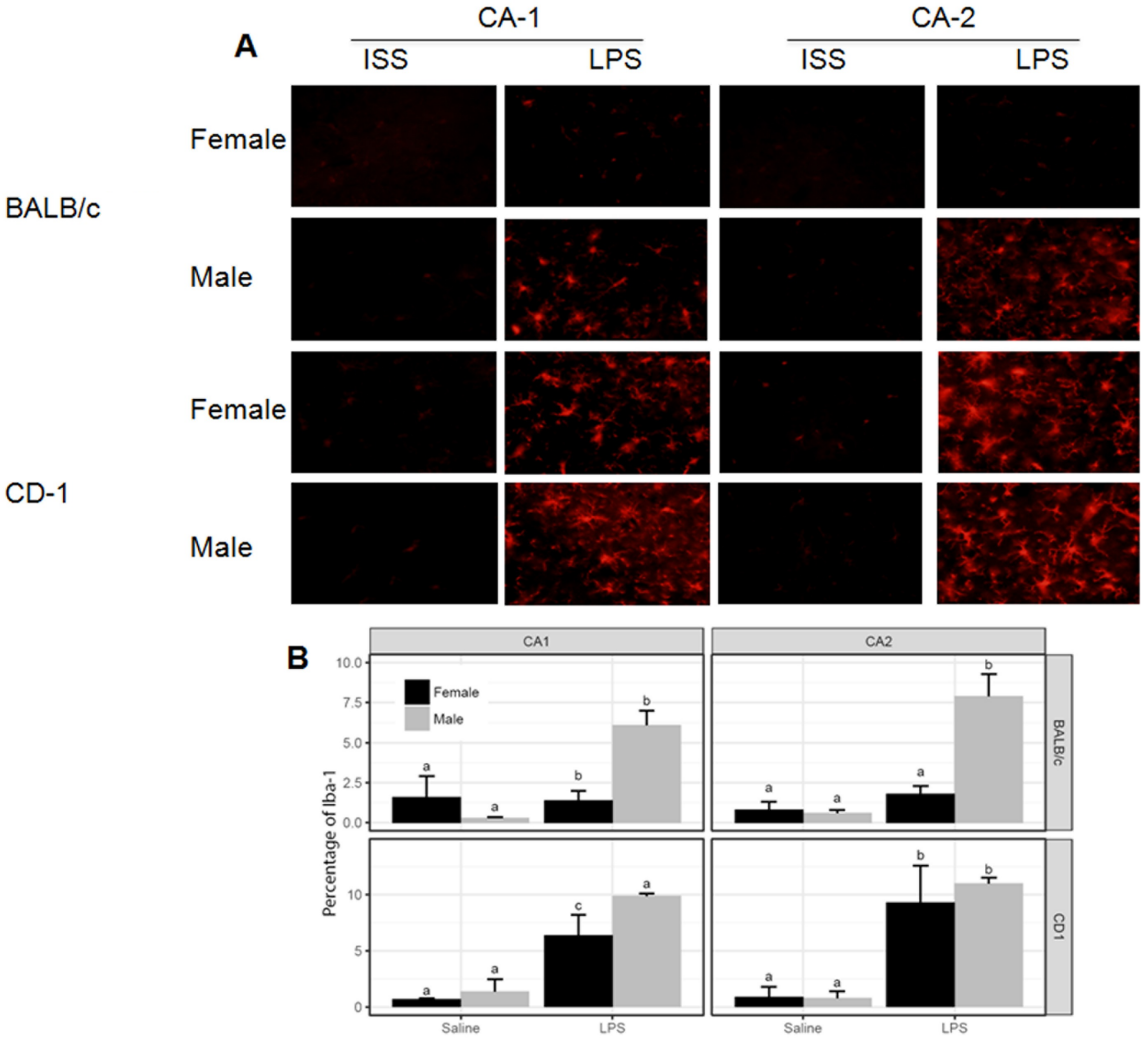
Immunohistological staining and quantification of Iba-1, 72 hrs after i.p. injection of LPS. A) Representative immunofluorescence of 30 μm coronal sections stained with anti-Iba-1 antibodies in two cornu ammonis (CA1 and CA2) regions. The pictures derive from saline and LPS-treated mice. B) Mean ± standard deviation of the percentage of Iba-1 using Image J software (National Institute of Health, Bethesda, MD, USA). The effects of the LPS in Iba-1 expression in each region were compared. Data labeled with the same letter are not significantly different from each other, whereas those with different letters are significantly different.

**Fig 3:**
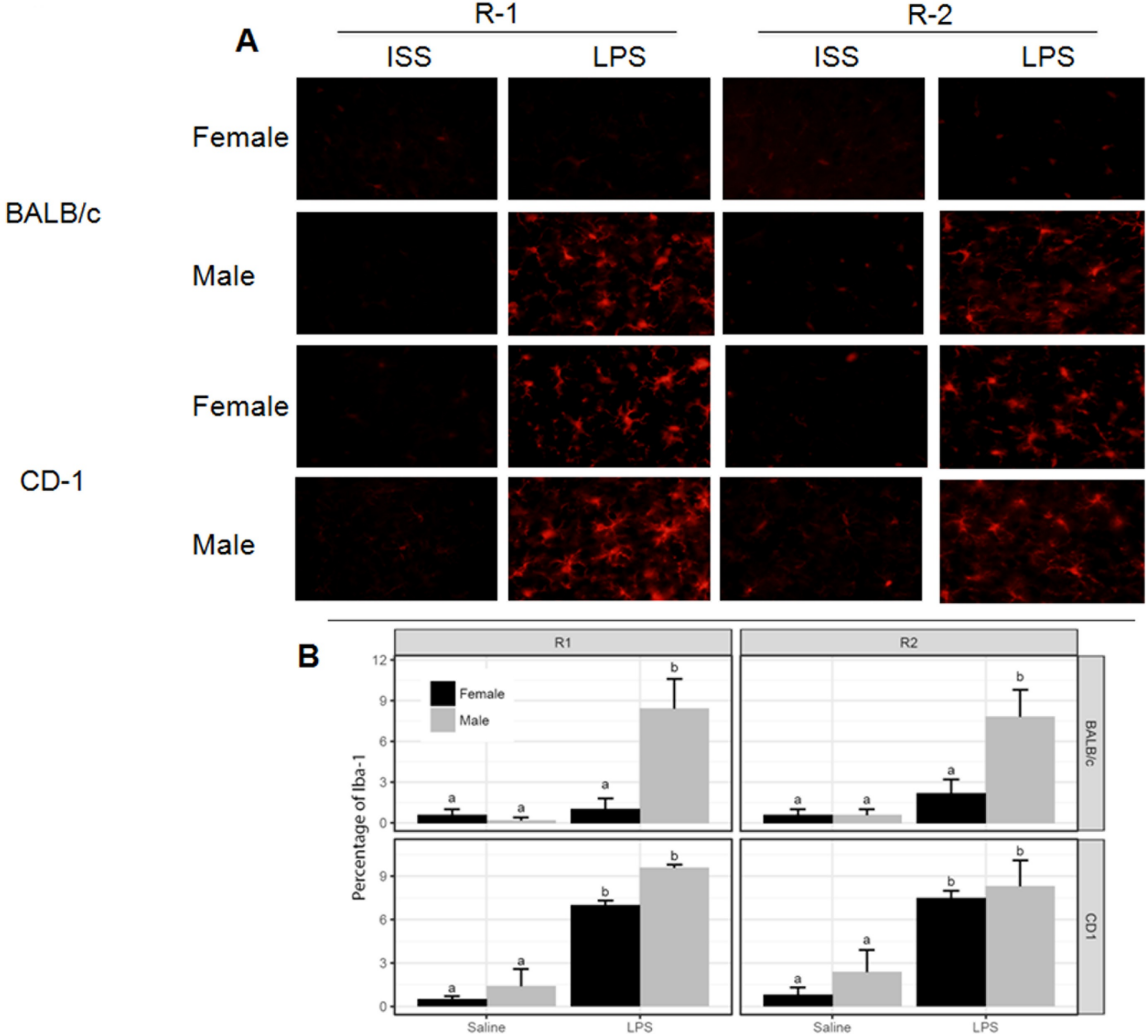
Immunohistological staining and quantification of Iba-1, 72 hrs after i.p. injection of LPS. **A)** Representative immunofluorescence of 30 μm coronal sections stained with anti-Iba-1 antibodies in two regions the cortex (R1 and R2). The pictures derive from saline and LPS-treated mice. **B)** Mean ± standard deviation of the percentage of Iba-1 using Image J software (National Institute of Health, Bethesda, MD, USA). The effects of the LPS in Iba-1 expression in each region were compared. Data labeled with the same letter are not significantly different from each other, whereas those with different letters are significantly different. No signal was detected in negative controls using TBS-2% BSA instead of the primary antibody.

No increment in the expression of Iba-1 and GFAP was detected 2 hours after administration of LPS (data not show). As Figs 2 and 3 show, a similar and significant increment in the expression of Iba-1 was observed in the four regions of the brain in both sexes of CD-1 as well as in BALB/c males 72 hours after administration of LPS. A lower expression of Iba-1 was observed in BALB/c female mice. GFAP not was significantly increased in LPS-treated mice regardless sex or strain (S2 Fig).

### Locomotor activity

**Fig 4:**
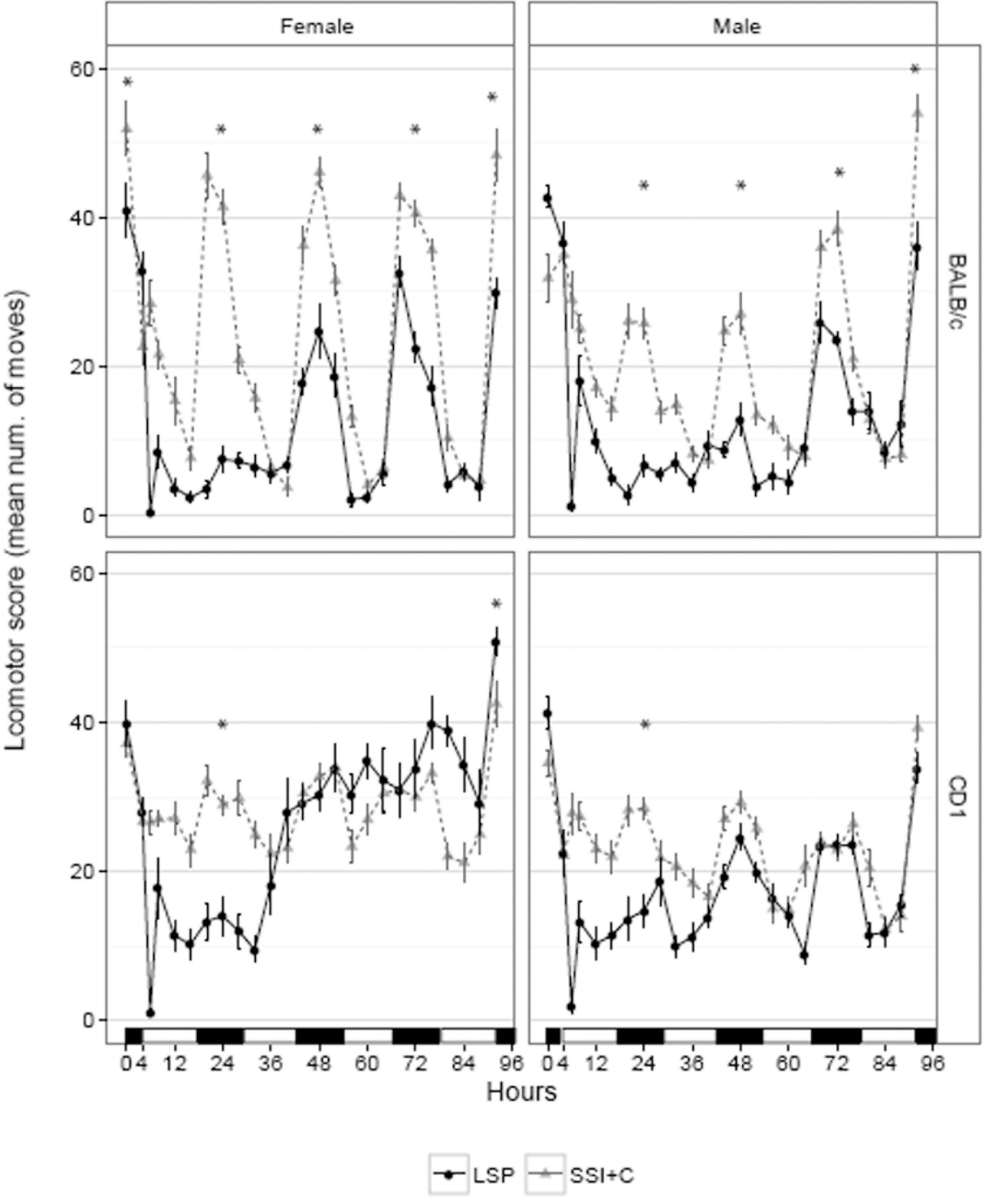
BALB/c and CD1 mice recovery of locomotor activity. Shapes mark mean locomotor activity measured at 24 hrs each day. Whiskers show SEM. Asterisks mark significant difference respect to saline and untreated controls.

Before treatment mice displayed stereotyped patterns of locomotion, showing little to no movement during the daylight hours, steadily increasing until they reach peak locomotor activity during the middle of the dark period (at the 24 hr mark). In general, female mice were more active than males. Also, CD-1 mice displayed higher levels of activity during the light period and lower levels than BALB/c during the dark period. The ISS injection did not modify this behavior. Locomotor activity of all mice significantly dropped after injection with LPS. A slow recuperation of locomotor activity in the days following LPS was observed, with varying rates of recovery depending on strain and sex. Female CD-1 mice reached normal levels of activity by 36 hrs after the LPS injection and males reach the normal locomotor activity at the third day (Fig 4). In contrast, BALB/c male and female mice did not reach the baseline levels displayed by controls during the 3 days studied (Fig 4).

## Discussion

The inflammatory status induced by peripheral injection of LPS on male and female BALB/c and CD-1 mice was defined based on the levels of IL-1β, IL-6 and TNF-α proinflammatory cytokines, as well as by the expression of activation markers Iba-1 and GFAP1 of microglia and astrocytes, respectively.

In the periphery, TNF-α, IL-1β and IL-6 were significantly increased in all mice two hours after LPS injection as previously reported [18], with differences depending on sex and strain. A clear sexual dimorphism was observed in the spleen cytokine levels. Males of both strains exhibited higher level of TNF-α. A higher level of IL-1β was observed in female BALB/c than in male mice. This sexual dimorphism is also observed for central levels of TNF-α in both strains and of IL-1β in CD-1. The increase in cytokines is accompanied by a substantial effect on the locomotor activity in both strains and sexes. It is known that TNF-α and IL1β, are cytokines that mediates symptoms of sickness such as anorexia, immobility and body weight loss [26].

A strain-sexual dimorphism in IL-6 has been also reported at different time-points up to 7 hrs following a LPS challenge, albeit under with a higher dose of LPS [27] than the used herein. In our study, mice were maintained for 72 hrs when male and female CD-1 mice recovered normal locomotion.

Different factors may underlie the strain-sexual dimorphism in the level of cytokines found in our study. LPS promotes the production of pro-inflammatory cytokines through the TLR4 signaling pathway, which in humans has found to be differentially expressed in neutrophils between sexes. Indeed, higher level of TLR4 is expressed in male neutrophils and consequently higher levels of TNF-α upon LPS stimulation were produced respect to those from female mice [28]. In addition it has been proposed that in mice, the genetic background modulates the TLR4 signaling pathway [29], and that there is a sexual dimorphism in the expression of TLR4, male mice showing higher expression of TLR4 in the cell surface of macrophages [25].

On the other hand, differences in the level of estradiol receptors and its signaling pathways could also differentially influence in the response to LPS between sexes. Certainly, increased estradiol levels in female rats protects them against an exacerbated inflammatory response [30–33].

Whilst cytokines were increased at two hrs, no changes in the activation status of microglia/macrophages or astrocytes were detected. In contrast, 72 hrs after LPS- injection central and peripheral cytokines had subsided and the expression of Iba-1 in all but female BALB/c mice was augmented. These findings are in line with the reports of a higher susceptibility of septic shock in males following bacterial sepsis, a pathological condition mimicked by LPS inflammatory model presented here [34]. The dimorphisms in the expression of cytokines produced as part of an inflammatory response could also in part explain the differential effect on the locomotor activity following LPS-treatment. Noticeable, before treatment, females were more active than males. LPS-treated BALB/c male and female mice did not reach the level of locomotor activity observed in controls, even three days after LPS. In contrast, CD-1 females recovered a normal level of activity faster than males. CD-1 females, also exhibited higher central levels of IL-6 at 2 hrs and Iba-1 at 72 hrs than BALB/c female. These results suggest that IL-6 may exhibit neuroprotective properties, as previously suggested in a rat model of ischemia, in which the administration of exogenous IL6 protects against neuronal death [35]. While CD-1 male mice also exhibited increased levels of IL-6, this increase was accompanied by increased central levels of TNF-α and IL-1β, two pro-inflammatory cytokines which may offset the apparent protective activity mediated by elevated levels IL-6. The reduction of expression of Iba-1, together with the failure to return to a normal level of activity may reflect the possible regulatory profile of the 72 hrs activated microglia that merit additional studies to ascertain factors involved.

Regarding other reported differences observed between strains, CD-1 appears to be less anxious than BALB/c mice when exposed to an open space test [36]. Moreover, humans experimentally treated with LPS display increased anxiety as well as increases in IL-6 and TNF-α, 2 to 3 hrs thereafter, being the changes in IL-6 more pronounced involved in their anxiety status [37]. Thus, it is probably that the sooner locomotion recovery of CD1 mice compared to BALB/c could be related with their lower anxiety status and their higher IL-6 concentrations, although other inflammatory mediators such as prostaglandins and leukotriens could be also involved with wellness.

In summary, our results show that acute systemic administration of LPS leads to peripheral and central increase of pro-inflammatory cytokines and microglia activation, in a strain and sex dependent manner with visible locomotor consequences.

## Supporting information

S1 Fig Cytokine levels in CD-1 and BALB/cAnN male and female mice 72 hours after LPS administration. BALB/cAnN and CD-1 male and female mice were treated with saline, LPS or no treated and 72 hrs brains and spleen of each mouse was recovered. †Mean (SD) of the level of cytokines (pg/mg of total protein). Cytokines concentration was measured in a soluble extract from the spleen or brains of each of 3 to 5 mice per group.
(PDF)

S2 Fig Immunohistological staining and quantification of GFAP, 72 hrs after i.p. injection of LPS. **A)** Representative immunofluorescence of 30 μm coronal sections stained with anti-GFAP antibodies in two cornu ammonis (CA1 and CA2) regions. The pictures derive from naive, saline and LPS-treated mice. **B)** Mean ± standard deviation of the percentage of GFAP, in two cornu ammonis (CA1 and CA2) regions and the cortex (R1 and R2) using Image J software (National Institute of Health, Bethesda, MD, USA). The effects of the LPS in Iba-1 expression in each region were compared. Data labeled with the same letter are not significantly different from each other, whereas those with different letters are significantly different.
(PDF)
