# Supplementary material for: Recovery from an acute systemic and central LPS-inflammation challenge is affected by mouse sex and genetic background
Source: PLoS One. 2018 Aug 22;13(8):e0201375. doi: 10.1371/journal.pone.0201375 (PMC6104912; doi:10.1371/journal.pone.0201375)
Supplement: S1 Fig — BALB/cAnN and CD-1 male and female mice were treated with saline, LPS or no treated and 72 hrs brains and spleen of each mouse was recovered. †Mean (SD) of the level of cytokines (pg/mg of total protein). Cytokines concentration was measured in a soluble extract from the spleen or brains of each of 3 to 5 mice per group. (PDF) [file pone.0201375.s001.pdf]

Supporting information

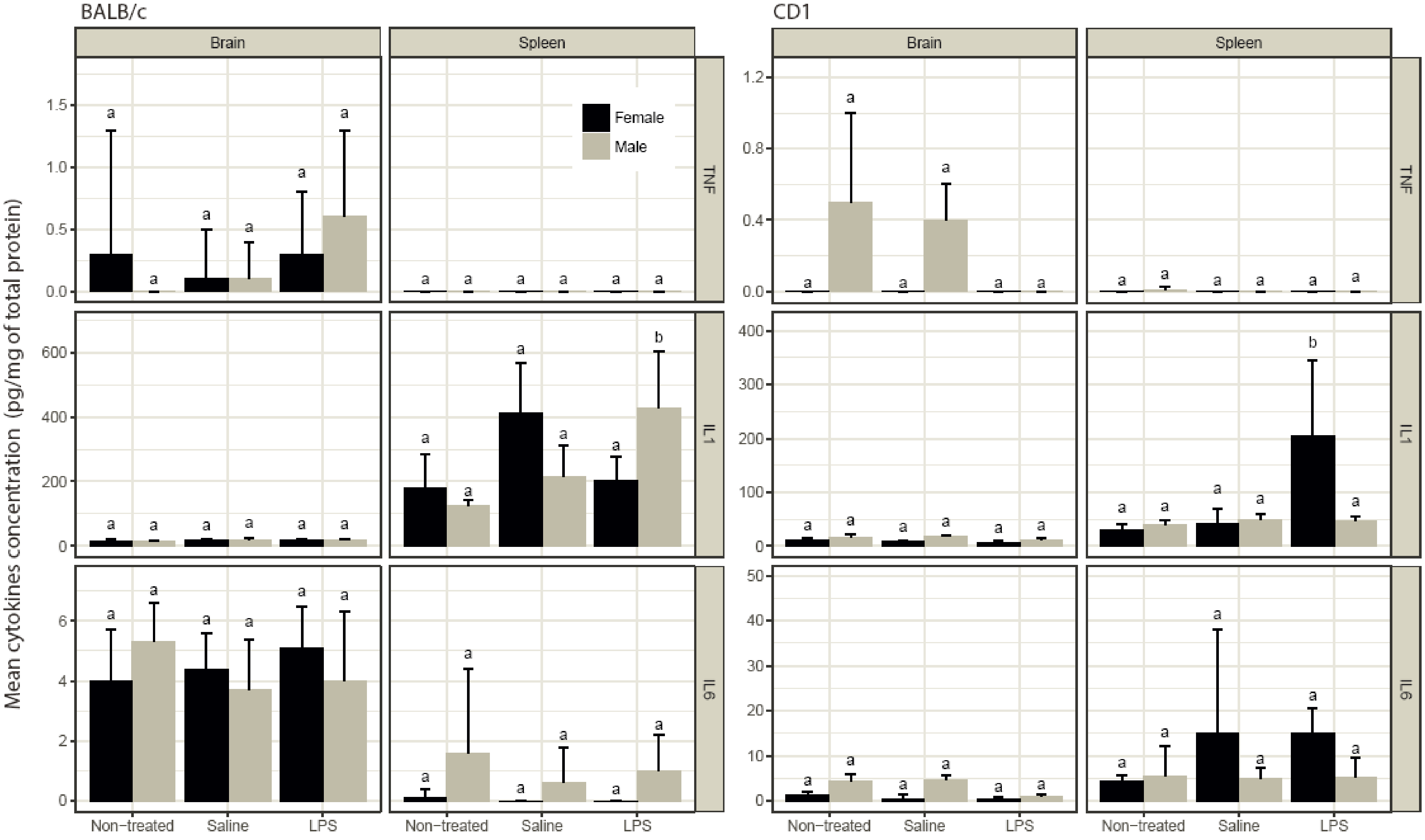

S1 Fig. Cytokine levels in CD-1 and BALB/cAnNmale and female mice 72 hours after LPS administration. BALB/cAnN and CD1 male and female mice were treated with saline, LPS or no treated and at 72 hrs brains and spleen of each mouse was recovered. †Mean (SD) of the level of cytokines (pg/mg of total protein). Cytokines concentration was measured in a soluble extract from the spleen or brains of each of 3 to 5 mice per group.
