# Supplementary material for: Recovery from an acute systemic and central LPS-inflammation challenge is affected by mouse sex and genetic background
Source: PLoS One. 2018 Aug 22;13(8):e0201375. doi: 10.1371/journal.pone.0201375 (PMC6104912; doi:10.1371/journal.pone.0201375)
Supplement: S2 Fig — A) Representative immunofluorescence of 30 μm coronal sections stained with anti-GFAP antibodies in two cornu ammonis (CA1 and CA2) regions. The pictures derive from naive, saline and LPS-treated mice. B) Mean ± standard deviation of the percentage of GFAP, in two cornu ammonis (CA1 and CA2) regions and the cortex (R1 and R2) using Image J software (National Institute of Health, Bethesda, MD, USA). The effects of the LPS in Iba-1 expression in each region were compared. Data labeled with the same letter are not significantly different from each other, whereas those with different letters are significantly different. (PDF) [file pone.0201375.s002.pdf]

Supporting information

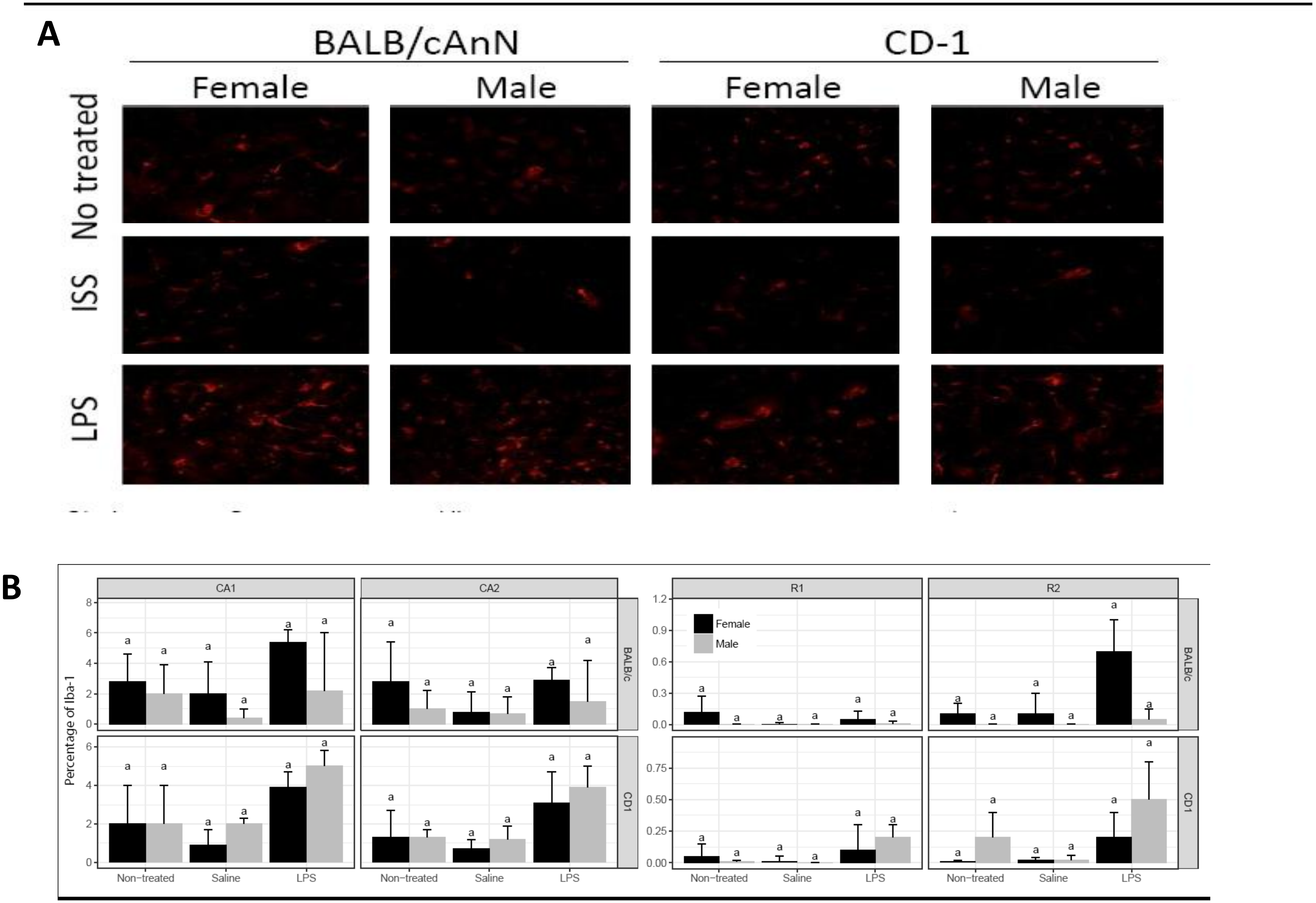

S2 Fig. Immunohistological staining and quantification of GFAP, 72 hrs after i.p. injection of LPS. **A)** Representative immunofluorescence of 30 µm coronal sections stained with anti-GFAP antibodies in two cornu ammonis (CA1 and CA2) regions. The pictures derive from naive, saline and LPS-treated mice. **B)** Mean ± standard deviation of the percentage of GFAP, in two cornu ammonis (CA1 and CA2) regions and the cortex (R1 and R2) using Image J software (National Institute of Health, Bethesda, MD, USA). The effects of the LPS in GFAP expression in each region were compared. Data labeled with the same letter are not significantly different from each other, whereas those with different letters are significantly different
